# Giant oral lipoma: a rare entity*

**DOI:** 10.1590/abd1806-4841.20165008

**Published:** 2016

**Authors:** José Burgos Ponce, Gustavo Zanna Ferreira, Paulo Sérgio da Silva Santos, Vanessa Soares Lara

**Affiliations:** 1 Bauru Dental School – University of São Paulo (USP) – Bauru (SP), Brazil; 2 Cesumar University Center (Unicesumar) – Maringá (PR), Brazil

**Keywords:** Lipoma, Mouth, Mouth mucosa, Mouth neoplasms

## Abstract

Lipomas are very common benign slow-growing soft tissue neoplasms composed of mature adipose tissue mostly diagnosed in the fifth decade of life. These tumors rarely present in the oral cavity, representing less than approximately 5% of all benign mouth tumors. They are usually less than 2cm in size and etiology remains unclear. We report a young male patient presenting with a giant lipoma in the buccal mucosa. Histopathology revealed a large area of mature fat cells consistent with conventional lipoma and an area of the mucosal lining of the lesion suggestive of *morsicatio buccarum*. In the present article, we emphasize the clinicopathological features and differential diagnosis of the disease.

## INTRODUCTION

Lipoma is a benign mesenchymal neoplasm, representing at least one-third of all benign tumors. It is most common on the trunk, shoulders, neck and axilla, being rare on the hands, lower legs or feet. Cases involving children are very uncommon.^1-3^ Lipomas are also rarely observed in the oral cavity.^3,4^ Oral lipoma (OL) represents less than approximately 5% of all benign mouth tumors, which occur in the buccal mucosa, parotid region, lips, submandibular region, tongue, palate, floor of the mouth and vestibule.^5-8^ Nevertheless, the buccal mucosa is the most commonly affected site.^5,7,8^ Although oral lipoma is commonly asymptomatic, it may interfere with speech and mastication.^3,4^

Histologically, lipomas can be classified as conventional lipoma or its variants: fibrolipoma, angiolipoma, spindle cell/pleomorphic lipoma, myxolipoma, chondroid lipoma, osteolipoma, myolipoma and intramuscular or infiltrating lipoma.^1,5^ Several cases of lipomas entrapping salivary gland tissue have been recently described and termed sialolipoma.^5^ Although clinical diagnosis of OL is usually apparent, salivary gland or other mesenchymal tumors should be included in the differential diagnosis for the disease.^3,4^

We report a young male with a large lipoma in the buccal mucosa and highlight the clinicopathological features and differential diagnosis of the disease.

## CASE REPORT

**Figure 1:**
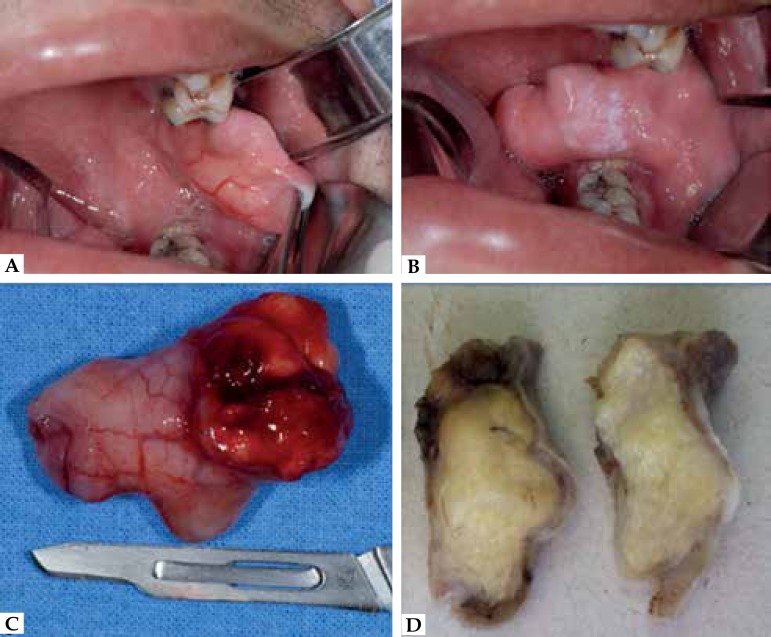
**A-B:** Clinical examination showing a nodular and pediculated mass in the left buccal mucosa region. **C-D:** Excised mass

**Figure 2:**
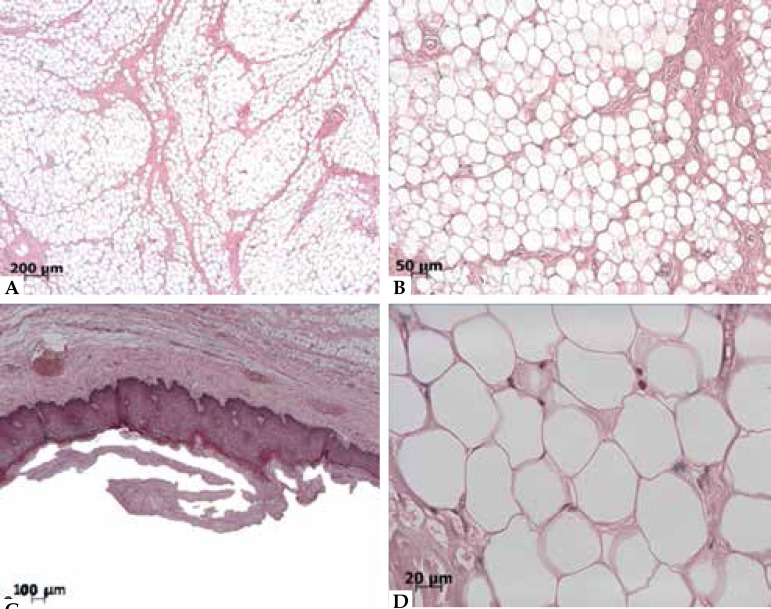
**A-B-D:** Histological examination showing mature adipocytes. **C:** Superficial layers suggestive of *morsicatio buccarum*

A 29-year-old male patient was referred to the Stomatology Clinics at Bauru Dental School – University of São Paulo – with a chief complaint of a 1–year painless movable mass in the left buccal mucosa region. Oral examination revealed a nodular and pedunculated mass with defined borders, regular contour and resilient consistency (Figure 1). The mucosa overlying the swelling was normal in color and appearance but showed irregular whitish areas consistent with occlusal trauma (Figure 2). Even though it was large, the lesion did not affect speech or chewing, but the patient acquired a parafunctional habit during tumor growth. Family history of the patient was unremarkable.

Clinical diagnosis was presumed to be a lipoma, and differential diagnosis included salivary gland tumor or other mesenchymal neoplasm. The patient signed a written consent form before the surgery. We resected the lesion off the adjacent muscle fibers of the buccinator under local anesthesia (Figure 1). The surgical specimen presented 5cm in diameter and did not float in saline.

Histopathologic examination showed fibrous connective tissue with a large area of mature fat cells, bundles of collagen fibers, blood vessels and a few mast cells. Peripherally, we observed scarce bundles of skeletal muscle tissue and mucous acini of salivary glands (Figure 2). Oral mucosa consisted of stratified squamous epithelium showing both hyper ortho- and parakeratotic layers with detachment of superficial layers suggestive of *morsicatio buccarum*. We also observed microbial biofilms on superficial layers (Figure 2). We diagnosed oral lipoma and established a 10-month follow-up. No recurrence was observed.

## DISCUSSION

Lipomas rarely occur in the oral cavity. When they are present, the most common place for their occurrence is the buccal mucosa, followed by the tongue.^3,4,5,6,8^ When localized at the floor of the mouth, lipomas may occasionally reach large size and interfere with speech, mastication, consequently requiring surgical intervention.^2,4,9^ Oral lipomas (OL) are rarely malignant, owing to the fact that lesions grow slowly and show a normal overlying mucosa and lack of nodal involvement.^1^

In this report, a large-size lesion was located in the left buccal mucosa. Although lipoma was our main diagnosis, we considered the possibility of different neoplasms and salivary gland tumors as differential diagnosis of the disease. Salivary gland adenomas, for example, can occur in the palate, lips and buccal mucosa, and some of them – like canalicular adenoma and duct papillomas – arise almost exclusively in minor salivary glands. Pleomorphic adenoma represents up to 70% of minor gland tumors.

Occasionally, lipomas may invade or grow between the muscles: the so-called intramuscular, infiltrating lipoma.^5,8^ In these cases, the tumors show greater recurrence rates after surgical treatment. On the other hand, although intraoral lipomas have no well-defined limits, they rarely recur. In the present case, we observed close contact between the lesion and the buccinator muscle during the surgery. Transurgical observations revealed the separation of the lesion from the deepest muscle tissue. The microscopic sections showed a well-circumscribed lesion, separated from the muscle bundles. Therefore, we discarded the diagnosis of intramuscular lipoma.

Histologically, lipoma cells cannot be distinguished from a herniated buccal fat pad. The herniation of a buccal fat pad presents as an expanding pedunculated mass emanating from the deep soft tissue in the buccal mucosa region, with a history of posttraumatic sudden onset.^10^ Differential diagnosis includes lipoma, foreign body granuloma, traumatic neuroma and salivary neoplasm. This lesion generally occurs in infants and children.^5^ Oral pathologists should pay special attentions to such events before treating patients.

Microscopic analysis of the present case also supports the clinical observation of occlusal trauma since it revealed an area consistent with *morsicatio buccarum*. Indeed, buccal mucosa is frequently traumatized, and the possible role of trauma in the growth of OL cannot be ruled out; nevertheless, no consensus exists regarding the pathogenesis of OL. ^1,5,10^ Fatty degeneration, heredity, hormonal basis, infection, metaphase of muscle cells, lipoblastic embryonic cell nest in origin, chronic irritation and trauma are possible theories that support the pattern of a lipoma.^10^

One aspect that caught our attention in the present case is that the lesion was very large, with 5cm at its largest diameter. Most lipomas on the body are smaller than 5cm and OL are usually less than 2cm.^3,4,6,7^ We could only find a few reported cases of this rare entity: large or giant OL.
